# Indole analogues decreasing the virulence of *Vibrio campbellii* towards brine shrimp larvae

**DOI:** 10.1111/1751-7915.14160

**Published:** 2022-11-05

**Authors:** Shanshan Zhang, Qian Yang, Tom Defoirdt

**Affiliations:** ^1^ Center for Microbial Ecology and Technology (CMET) Ghent University Ghent Belgium

## Abstract

Indole signalling has been proposed as a potential target for the development of novel virulence inhibitors to control bacterial infections. However, the major structural features of indole analogues that govern antivirulence activity remain unexplored. Therefore, we investigated the impact of 26 indole analogues on indole‐regulated virulence phenotypes in *Vibrio campbellii* and on the virulence of the bacterium in a gnotobiotic brine shrimp model. The results demonstrated that 10 indole analogues significantly increased the fluorescence of indole reporter strain *Vibrio cholerae* S9149, 21 of them decreased the swimming motility of *V. campbellii*, and 13 of them significantly decreased the biofilm formation of *V. campbellii*. Further, we found that 1‐methylindole, indene, 2,3‐benzofuran, thianaphthene, indole‐3‐acetonitrile, methyl indole‐3‐carboxylate, 3‐methylindole, and indole‐2‐carboxaldehyde exhibited a significant protective effect on brine shrimp larvae against *V. campbellii* infection, resulting in survival rates of challenged brine shrimp above 80%. The highest survival of shrimp larvae (98%) was obtained with indole‐3‐acetonitrile, even at a relatively low concentration of 20 μM. Importantly, the indole analogues did not affect bacterial growth, both in vitro and in vivo. These results indicate the potential of indole analogues in applications aiming at the protection of shrimp from vibriosis.

## Graphical abstract

Indole signaling controls the virulence of vibrios. In this study we identify indole analogues with a higher activity than indole, protecting brine shrimp from Vibrio campbellii.

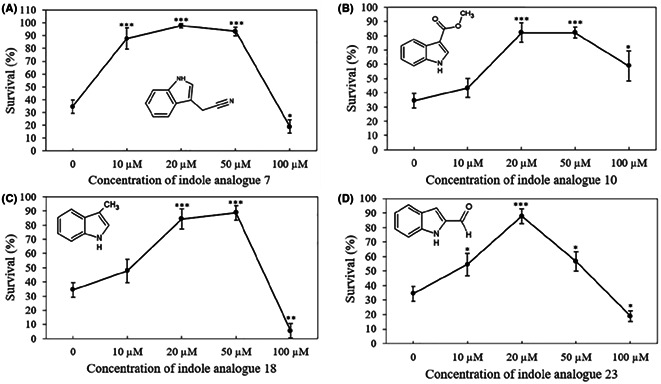

## INTRODUCTION

Aquaculture is the world's fastest growing food production sector as it is critically important for the nutrition and employment of millions of people (FAO, 2019). Shrimp is the second most traded seafood in the world in monetary terms, and shrimp farming is one of the major aquaculture sectors (Kumar & Engle, 2016; Sivaraman et al., 2018). Bacterial diseases such as vibriosis caused by various vibrios are one of the most important threats to sustainable aquaculture, causing tremendous economic losses (Novriadi, 2016). Luminescent shrimp disease caused by luminescent *Vibrio harveyi* and closely related species is a serious problem in shrimp farming, where the mortality can be as high as 100% (Diggles et al., 2000; Kumar et al., 2021; Lavilla‐Pitogo et al., 1998; Yatip et al., 2018). Vibrios belonging to the *Harveyi* clade, such as *V. harveyi* and closely related species such as *V. alginolyticus*, *V. campbellii* and *V. parahaemolyticus*, are important pathogens causing vibriosis outbreaks in aquaculture all over the world (Thompson et al., 2004).

In order to control these bacterial diseases, farmers have used antibiotics in the aquaculture environment to protect their animals. However, the frequent use of antibiotics has resulted in the emergence of multidrug‐resistant strains, and has led to an international health crisis of aquatic animals and humans (Rasul & Majumdar, 2017; Scott et al., 2019). Therefore, there is an urgent need for a truly novel strategy to control bacterial diseases in aquaculture without antibiotics (Defoirdt et al., 2011). Among the newly developed therapeutic strategies, antivirulence therapy has been deemed to be a promising alternative. Instead of killing pathogens, antivirulence therapy aims at disarming pathogens by inhibiting bacterial virulence, thereby preventing the pathogens from attacking their host (Defoirdt, 2018; Dickey et al., 2017).

**FIGURE 1:**
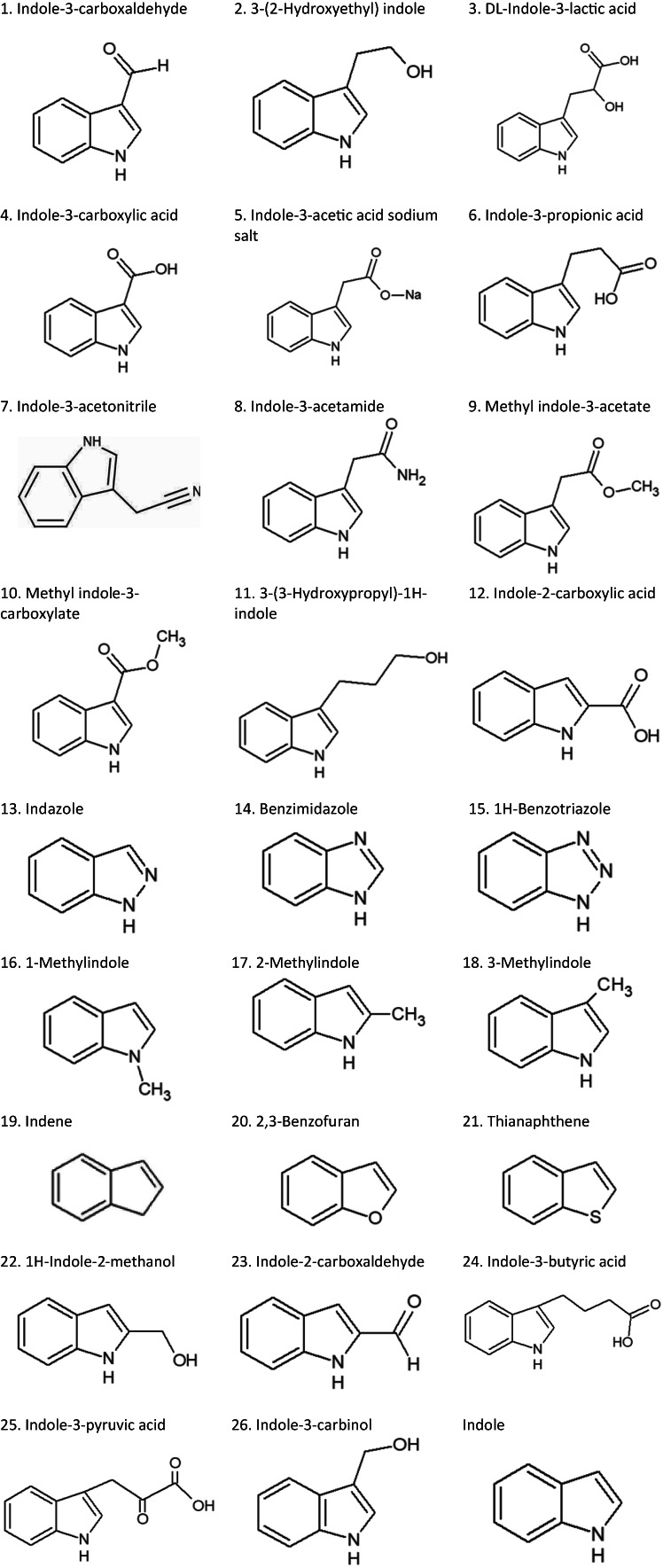
Indole analogues used in this study. Indole analogues used in this study

Indole signalling has been proposed as a potential target for the development of novel antivirulence compounds because of its impact on virulence factor production in various bacterial pathogens (Lee et al., 2015; Melander et al., 2014). Indole is produced from tryptophan by tryptophanase (TnaA) in a large number of Gram‐positive and Gram‐negative bacterial species, including *V. campbellii* (Deeley & Yanofsky, 1981; Lee & Lee, 2010; Yang et al., 2017). It controls diverse aspects of bacterial physiology, such as spore formation (Kim et al., 2011), plasmid stability (Chant & Summers, 2007), drug resistance (Vega et al., 2012; Zhang et al., 2020), biofilm formation (Lee, Jayaraman, & Wood, 2007; Zhang et al., 2021), and virulence (Bommarius et al., 2013; Hirakawa et al., 2009). Interestingly, we previously found that indole decreases the virulence of vibrios belonging to the Harveyi clade towards gnotobiotic brine shrimp (*Artemia franciscana*) larvae and conventionally reared giant river prawn (*Macrobrachium rosenbergii*) larvae (Yang et al., 2017; Zhang et al., 2021). It was also shown that the major indole‐controlled virulence‐related phenotypes in these bacteria were flagellar motility and biofilm formation both of which are linked to the virulence of the bacterium (Yang et al., 2017). However, there is a limitation to the practical use of indole as an antivirulence drug because it showed to be toxic to the cultured animals at a concentration (200 μM) that was only twofold higher than the concentration needed to obtain protection from vibriosis (100 μM). Therefore, more potent and/or less toxic indole analogues are needed. In the present study, we explored the impact of a set of 26 indole analogues (Figure 1) on flagellar motility and biofilm formation in *V. campbellii* and on its virulence towards gnotobiotic brine shrimp larvae.

## EXPERIMENTAL PROCEDURES

### Bacterial strains, culture conditions, and chemicals

Two strains were used in this study: *Vibrio campbellii* BB120 (ATCC BAA‐1116) and *Vibrio cholerae* strain S9149 (SIO *∆lacZ ∆tnaA ∆vpsL::lacZ*). The latter strain produces beta‐galactosidase (LacZ) in the presence of indole (Mueller et al., 2009). The bacteria were cultured in Luria–Bertani medium containing 35 g/L of sea salt (LB_35_) at 28°C under constant agitation (100 min^−1^). Cell densities were measured spectrophotometrically at 600 nm. Indole analogues were purchased from Sigma‐Aldrich. Indole analogues are listed in Figure 1. They were dissolved in methanol at 10, 20, 50, 100 and 200 mM, respectively. In all experiments, all treatments received the same volume of methanol (0.1% v/v).

### Determination of the impact of the analogues on the growth curve of *V. campbellii*

To investigate the effect of the indole analogues on the growth of *V. campbellii* BB120, overnight grown cells were inoculated into fresh LB_35_ medium at an initial OD_600_ of 0.01. Indole analogues were added at 0, 100 or 200 μM, respectively. Then, 200 μl aliquots of these suspensions were pipetted into the wells of a polystyrene 96‐well plate and cultured at 28°C for 24 h. The OD_600_ of each sample was measured every hour with a Tecan Infinate M200Pro plate reader (Tecan). Growth curves were determined for three independent cultures.

### Determination of the impact of the analogues on the beta‐galactosidase activity of *V. cholerae* indole reporter strain S9149

For this experiment, the *V. cholerae* indole reporter strain S9149 was used (Mueller et al., 2009). This strain contains a *vpsL::lacZ* fusion as well as a deletion of *tnaA* and *lacZ*. As the *vpsL* gene is induced by indole, this means that this strain will produce beta‐galactosidase activity in the presence of exogenous indole. The FluoReporter lacZ/Galactosidase Quantitation Kit (Thermo Fisher) was used to measure the beta‐galactosidase activity of *V. cholerae* S9149 according to the manufacturer's instructions. The strain was grown overnight and transferred (5% v/v) to fresh LB_35_ with indole at concentrations of 10, 20, 40, 60, 80, 100 and 200 μM to construct a standard curve. The same protocol listed above was performed with the indole analogues, which were tested at 200 μM only. The cultures were further incubated at 28°C with shaking for 10 h. One ml culture from each sample was collected and washed twice with phosphate‐buffered saline (PBS). Then, 350 μg of 0.1 mm glass beads was added. The samples were shocked with a PowerLyzer™ 24 Bench Top Bead‐Based Homogenizer (Qiagen). After that, 10 μl cell extract of each sample was collected and 20‐fold diluted. Ten μl aliquots of the diluted samples were added into black microplate wells, and 10 μl reaction buffer was used as a blank. Meanwhile, 100 μl of CUG working buffer was added to each well and incubated for 30 min at room temperature. Finally, 50 μl of stop buffer was added to each well to terminate the reaction, and the fluorescence of the solutions was measured with a Tecan Infinite 200 microplate reader fitted with an excitation filter centered at 390 nm and an emission filter at 460 nm.

### Determination of swimming motility

The swimming motility was determined on LB_35_ soft agar plates containing 0.2% agar (Yang & Defoirdt, 2015). *Vibrio campbellii* BB120 was grown overnight in LB_35_ medium, and diluted to an OD_600_ of 1. The LB_35_ soft agar was cooled down to approximately 50°C after autoclaving. Then, different indole analogues were added at a concentration of 200 μM. The agar was poured into petri plates and left open at room temperature for 15 min. Aliquots of 5 μl of the bacterial suspensions were added to the center of soft agar plates (six replicate plates per treatment). The plates were incubated upright at 28 °C and the motility halo diameters were measured after 1 day.

### Determination of biofilm levels

Biofilm formation was quantified by crystal violet staining, as described previously (Stepanovic et al., 2007). Briefly, an overnight culture of *V. campbellii* BB120 was diluted to an OD_600_ of 0.1, and indole analogues were added at 200 μM. Then, 200 μl aliquots of these suspensions were pipetted into the wells of a polystyrene 96‐well plate and cultured without agitation at 28°C for 24 h. After that, the wells were washed three times with PBS to remove unattached cells. Subsequently, the remaining attached bacteria were fixed with 200 μl methanol per well for 20 min, after which the methanol was removed and the plates were air‐dried. Then, biofilms were stained for 15 min with 200 μl per well of a 1% crystal violet solution. Plates were then rinsed with running water until the washings were free of the stain. After the plates were air‐dried, bound crystal violet was dissolved in 200 μl of 95% ethanol per well for 30 min, and absorbance was measured at 570 nm with a Tecan Infinate M200Pro plate reader. Sterile medium was served as a negative control, and the reported values are blank‐corrected.

### Axenic hatching of brine shrimp larvae

Five hundred milligrams of high‐quality hatching cysts of *Artemia franciscana* (EGVR Type; INVE Aquaculture) were hydrated in 45 ml of filter‐sterilised tap water for 1 h. Sterile cysts and larvae were obtained by decapsulation according to Marques et al. (2005). In brief, 1.65 ml of NaOH (32%) and 25 ml of NaOCl (50%) were added to the hydrated cyst suspension to facilitate decapsulation. The process was stopped after 2 min by adding 35 ml of Na_2_S_2_O_3_ (10 g/L). Filtered (0.22 μm) aeration was provided during the reaction. The decapsulated cysts were washed with filtered and autoclaved artificial seawater (containing 35 g/L of instant ocean synthetic sea salt; Aquarium Systems). The cysts were resuspended in a bottle containing 1 L of filtered and autoclaved synthetic seawater and hatched for at least 28 h at 28°C with aeration and constant illumination (2000 lux). The sterility of the cysts was verified by inoculating 1 ml of culture water into 9 ml of LB_35_ and incubating at 28°C for 24 h. After 28 h of hatching, batches of 30 larvae were counted and transferred into fresh, sterile 50 ml tubes containing 30 ml of filtered and autoclaved seawater. Finally, the tubes were put on a rotor (four rotations per min) and kept at 28°C. All manipulations were performed in a laminar flow hood in order to maintain sterility of the cysts and larvae.

### Brine shrimp challenge tests

The impact of selected indole analogues on the virulence of *V. campbellii* BB120 was determined using a standardised challenge test with gnotobiotic brine shrimp larvae, as described by Defoirdt et al. (2005). *V. campbellii* BB120 was added to the brine shrimp rearing water at 10^6^ CFU/ml. The indole analogues (at the concentrations indicated in the Results) were added into the brine shrimp rearing water either at the start of the experiment or after 24 h of incubation. A suspension of autoclaved LVS3 bacteria (Verschuere et al., 1999) in filtered and autoclaved artificial seawater was added into all of the cultures as feed at the start of the challenge test at 10^7^ cells/ml. Brine shrimp cultures to which only methanol and autoclaved LVS3 bacteria were added, were used as controls. The survival of the larvae was counted 48 h after the addition of the pathogen. Each treatment was carried out in triplicate. In each test, the sterility of the control treatments was checked at the end of the challenge by inoculating 1 ml of rearing water of the control treatment (no pathogens added) to 9 ml of LB_35_ and incubating the mixture for 2 days at 28°C. The concentrations of *V. campbellii* BB120 in the brine shrimp rearing water at the end of the experiment were determined by plate counting on LB_35_ agar.

## RESULTS AND DISCUSSION

### Impact of the indole analogues on the growth of *V. campbellii*

In the first experiment, we determined the impact of the indole analogues on the growth of *V. campbellii* as we aimed for analogues that affect the virulence, but not the viability of the pathogen. None of the indole analogues (at a concentration of up to 200 μM) inhibited the growth of *V. campbellii* (Figure S1).

### Impact of the indole analogues on beta‐galactosidase activity of indole reporter strain *V. cholerae* S9149

**FIGURE 2:**
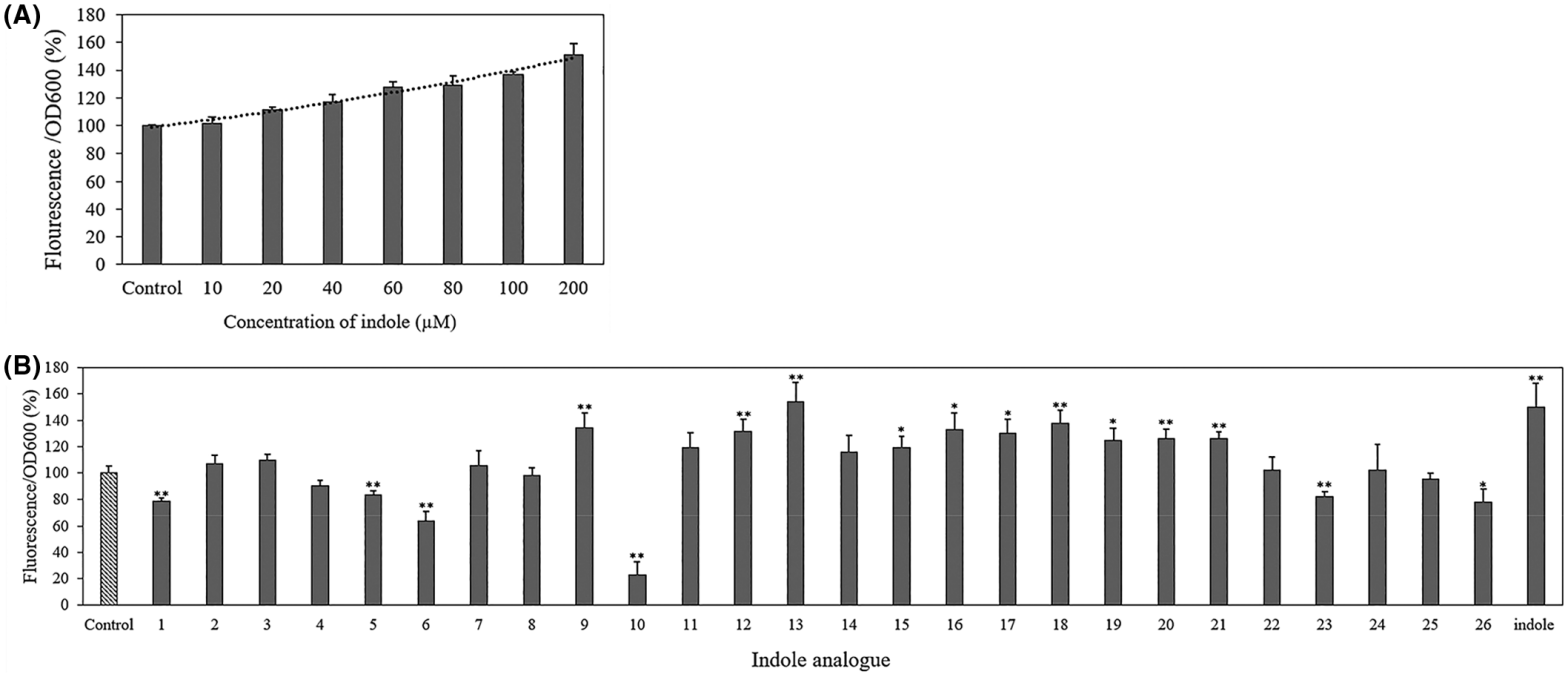
(A) Beta‐galactosidase activity (fluorescence/OD600) of *Vibrio cholerae* S9149 as function of the concentration of indole. (B) Impact of 200 μM of the indole analogues on the beta‐galactosidase activity of *V. cholerae* S9149. Control refers to untreated *V. cholerae* S9149. The fluorescence in the control treatment was set at 100% and the other treatments were normalised accordingly. Asterisks indicate significant differences when compared to the control treatment (independent‐sample *T*‐test); *p < 0.05, **p < 0.01 and ***p < 0.001

In order to select indole analogues for in vivo challenge tests, we performed three in vitro tests in which indole‐regulated activity was determined. A first experiment involved *V. cholerae* strain S9149. This strain is a *lacZ* and *tnaA* deletion mutant containing *lacZ* under the control of the *vpsL* promoter (which is activated by indole in *V. cholerae*; Mueller et al., 2009). This strain thus shows beta‐galactosidase activity in response to exogenously added indole (or a stimulating indole analogue). In order to determine beta‐galactosidase activity of *V. cholerae* S9149, 3‐carboxyumbelliferyl β‐d‐galactopyranoside was used, which produces the blue‐fluorescent hydrolysis product 7‐hydroxycoumarin‐3‐carboxlic acid on action of beta‐galactosidase. As shown in Figure 2A, indole increased the fluorescence of *V. cholerae* S9149, and the effect was proportional to the concentration of indole. In order to select indole analogues with a similar activity as indole, *V. cholerae* S9149 was cultured in 200 μM of each of the different analogues. The results indicated that the fluorescence of *V. cholerae* S9149 was significantly increased by indole analogues 9, 12, 13, and 15–21 (Figure 2B), although for most analogues the effect was smaller than that of indole at the same concentration. This indicates that the determination of the impact of indole analogues on the beta‐galactosidase activity is not the most sensitive way to identify active indole analogues.

### Impact of the indole analogues on swimming motility and biofilm formation of *V. campbellii*

**FIGURE 3:**
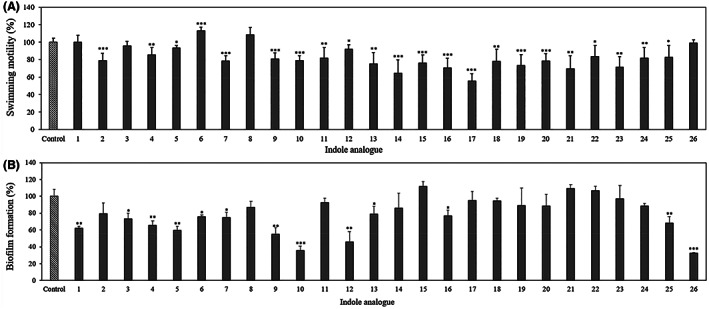
Impact of 200 μM of indole analogues on swimming motility (A) and biofilm formation (B) of *V. campbellii* BB120. Swimming motility halos were measured after 24 h incubation on soft agar. Biofilm formation on polystyrene microtitre plates was determined by crystal violet staining. *Vibrio campbellii* BB120 cultured with the same volume of methanol was used as a control. The swimming motility and biofilm formation in the control treatment was set at 100% and the other treatments were normalised accordingly. Error bars represent the standard deviation of six replicates for swimming motility and three replicates for biofilm formation. Asterisks indicate significant differences when compared with the control treatment without indole analogue (independent‐samples T‐test); *p < 0.05, **p < 0.01 and ***p < 0.001

To explore the impact of the indole analogues on indole‐controlled virulence related phenotypes of *V. campbellii*, swimming motility and biofilm formation were determined. Both of these phenotypes are decreased in the presence of indole in *V. campbellii* (Yang et al., 2017). The results of the swimming motility assay showed that indole analogues 2, 4, 5, 7, and 9–25 significantly decreased the swimming motility (Figure 3A). However, the impact of the indole analogues on motility was relatively modest, as only analogue 17 decreased the motility of *V. campbellii* to less than 60% of the control treatment. It has been reported that bacterial motility can be affected by several kinds of indole analogues. For example, 7‐hydroxyindole (Lee et al., 2009) and 7‐fluoroindole (Lee, Kim, Cho, et al., 2012) inhibited the swarming motility of *Pseudomonas aeruginosa*; and 6‐fluoroindole and 7‐methylindole decreased the swimming and swarming motility of *Serratia marcescens* (Sethupathy et al., 2020).

In the biofilm experiment, biofilm levels were significantly decreased in the presence of indole analogues 1, 3–7, 9, 10, 12, 13, 16, 25, and 26 (Figure 3B). The strongest effect was observed for analogues 9, 10, 12 and 26 (decreasing the biofilm formation of *V. campbellii* down to <60% of the control treatment). Indole has been reported to inhibit biofilm formation in 10 strains of Harveyi clade vibrios (Zhang et al., 2021). Further, indole analogues like 7‐fluoroindole (Lee, Kim, Cho, et al., 2012) and 3‐indolylacetonitrile (Lee et al., 2011) decreased biofilm formation of *P. aeruginosa*; indole‐3‐acetaldehyde decreased biofilm formation of *Escherichia coli* (Lee, Kim, Kim, et al., 2012); and 6‐fluoroindole and 7‐methylindole which decreased swimming and swarming motility also showed the ability to inhibit biofilm formation in *S. marcescens* (Sethupathy et al., 2020). Finally, 7‐hydroxyindole inhibited biofilm formation in *E. coli* while it increased biofilm formation in *P. aeruginosa* (Lee, Bansal, et al., 2007). It is remarkable that both biofilm formation and motility were blocked by the indole anlogues (and also by indole; Yang et al., 2017) as both phenotypes are inversely regulated by c‐di‐GMP in many bacteria (Hengge, 2009). In case of *V. campbellii* this has not been shown yet, and even if the same would hold for *V. campbellii* this does not exclude the possibility that both phenotypes are affected in the same way by another regulatory mechanism. At this moment, however, such a mechanism has not been identified yet.

### Impact of the indole analogues on the virulence of *V. campbellii* in the gnotobiotic brine shrimp (*Artemia franciscana*) model

**FIGURE 4:**
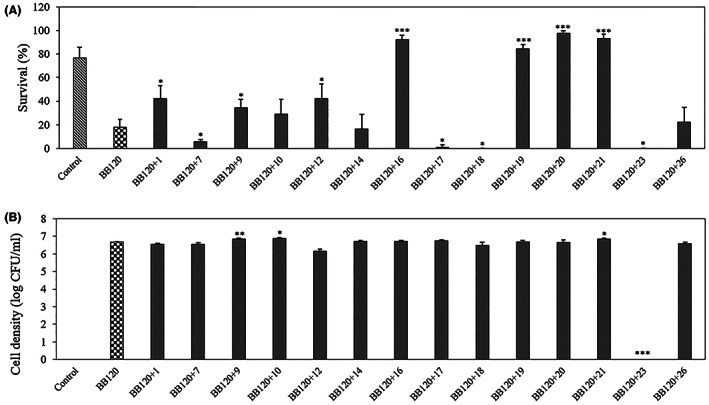
(A) Percent survival of brine shrimp (*Artemia franciscana*) larvae after 2 days of challenge with *V. campbellii* BB120 and 200 μM of selected indole analogues. *Vibrio campbellii* BB120 and the indole analogues were added to the brine shrimp rearing water at the start of the experiment. (B) Density of *V. campbellii* BB120 with or without 200 μM of selected indole analogues in the brine shrimp larvae rearing water after 2 days of challenge. Error bars represent the standard deviation of three shrimp cultures. Control refers to brine shrimp cultures without addition of *V. campbellii* BB120 or indole analogue that were treated in the same way as the other cultures. Asterisks indicate significant differences when compared to the treatment of BB120 without indole analogue (independent‐samples T‐test); *p < 0.05, **p < 0.01 and ***p < 0.001

According to the results obtained in the previous experiments, we selected indole analogues 1, 7, 9, 10, 12, 14, 16, 17, 18, 19, 20, 21, 23, and 26 for brine shrimp challenge tests. The indole analogues were added to the brine shrimp rearing water together with *V. campbellii* BB120 at the start of experiment. After 2 days of culture, the survival of the larvae was (almost) zero in the presence of analogues 7, 17, 18 and 23, suggesting that these compounds are toxic at the concentration used. We currently do not know why these compounds are toxic at this concentration. Comparing to the brine shrimp larvae cultured in the presence of *V. campbellii* BB120 without indole analogues (18% survival), the survival of brine shrimp larvae was strongly improved by indole analogues 16, 19, 20 and 21, as it reached 92%, 84%, 98% and 93% respectively, which was similar to or even better than the survival obtained for unchallenged larvae (77%) (Figure 4A). To determine the density of *V. campbellii* BB120 in the brine shrimp larvae rearing water after 2 days of challenge, we collected the rearing water and plated it on LB_35_ agar. The results indicated that there were no bacteria in the control and in the brine shrimp larvae rearing water with indole analogue 23. For the other analogues, the bacterial cell densities were the same as the density obtained in the treatment without indole analogues (Figure 4B).

**FIGURE 5:**
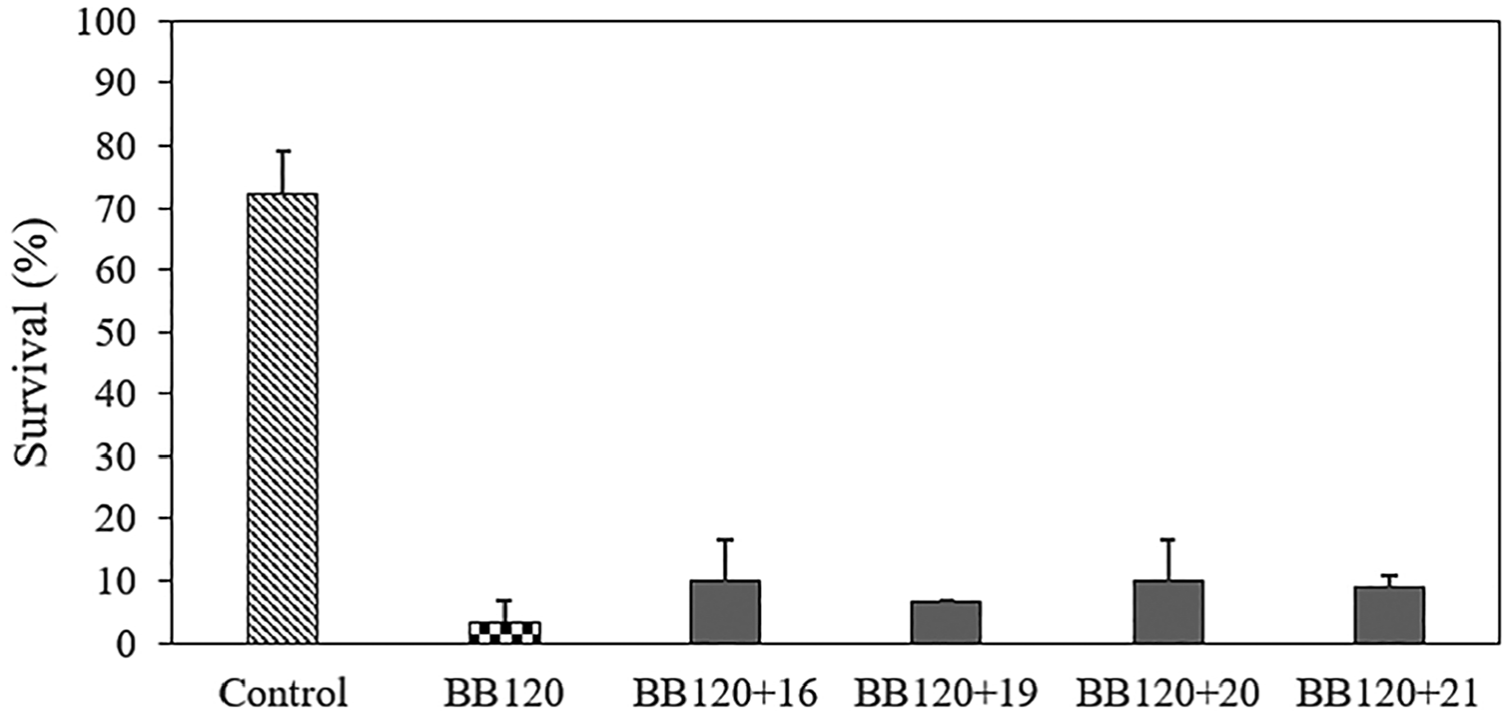
Percent survival of brine shrimp (*Artemia franciscana*) larvae after 2 days of challenge with *V. campbellii* BB120 and 200 μM of selected indole analogues. *Vibrio campbellii* BB120 was added to the brine shrimp rearing water at the start of the experiment, and the selected indole analogues were added after 1 day. Error bars represent the standard deviation of three shrimp cultures. Control refers to brine shrimp cultures without addition of *V. campbellii* BB120 that were treated in the same way as the other cultures.

Based on the previous experiment, we selected indole analogues 16 (1‐methylindole), 19 (indene), 20 (2,3‐benzofuran) and 21 (thianaphthene) for more detailed experiments. In order to determine whether in addition to a preventive action, these compounds also showed some curative action, we added the indole analogues into the rearing water 1 day after the addition of *V. campbellii* BB120. As shown in Figure 5, the survival of brine shrimp larvae was below 10% in all treatments, indicating that the indole analogues do not have a curative action against *V. campbellii*. This might indicate that the virulence factors that are controlled by indole are required during the first stages of infection and that indole (or indole analogues) are unable to protect brine shrimp once a certain stage of infection has been passed.

**FIGURE 6:**
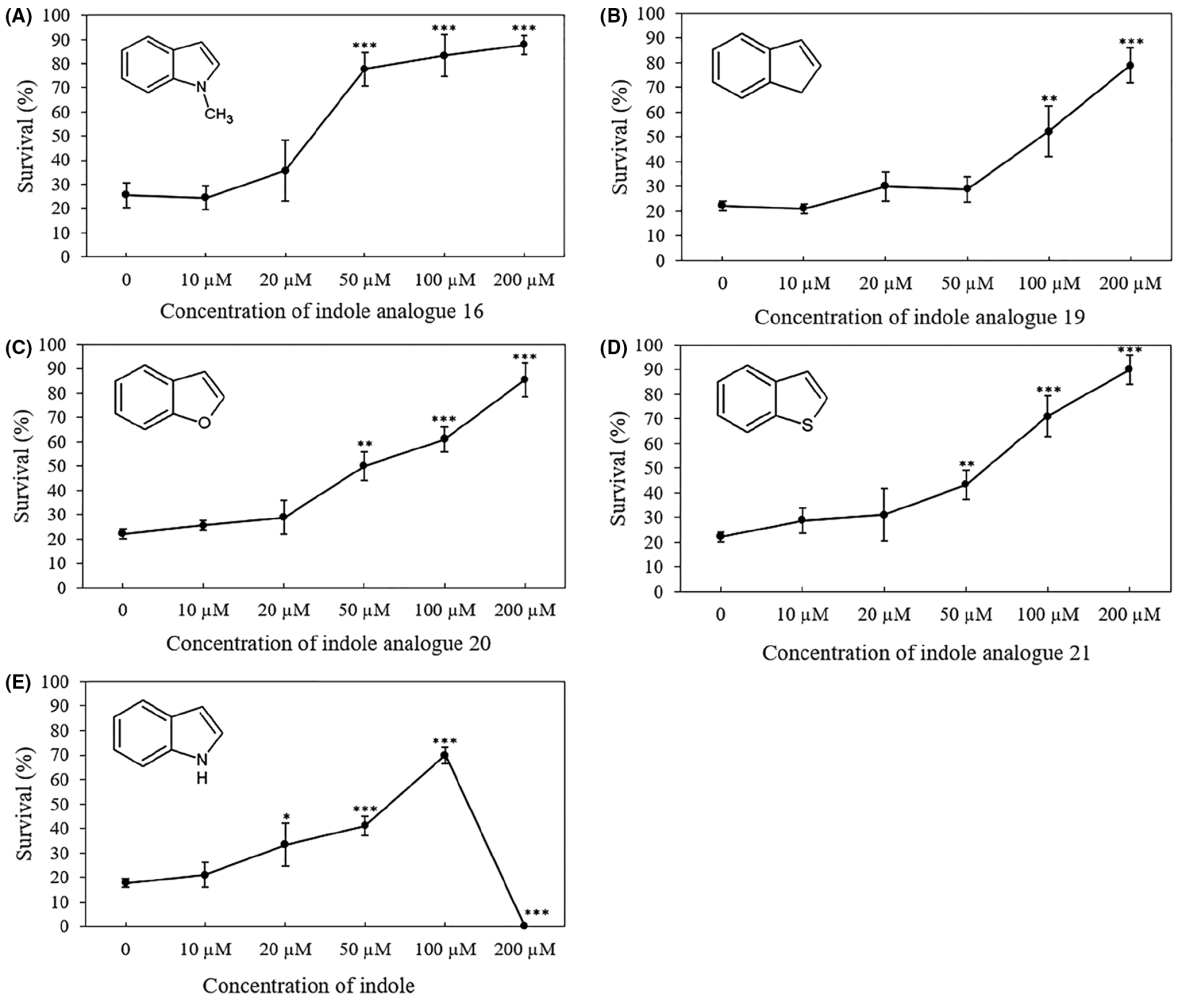
Percent survival of brine shrimp (*Artemia franciscana*) larvae after 2 days of challenge with *Vibrio campbellii* BB120 and different concentrations of selected indole analogues 16 (A), 19 (B), 20 (C), 21 (D) and indole (E). Indole and the indole analogues were added to the brine shrimp rearing water at the start of the experiment. Error bars represent the standard deviation of three shrimp cultures. The survival of unchallenged larvae that were otherwise treated in the same way as challenged larvae was 80% ± 6%. Asterisks indicate significant differences when compared to the treatment without indole analogue (independent‐samples *T*‐test); *p < 0.05, **p < 0.01 and ***p < 0.001

We further wanted to know whether these analogues would also offer preventive protection against *V. campbellii* at lower concentrations than 200 μM. To this end, we set up a challenged test in which we determined the impact of 10, 20, 50, 100 and 200 μM of the selected analogues on the survival of brine shrimp larvae. For comparison, we also included indole in this experiment. The results indicated that the survival of the brine shrimp larvae was proportional to the concentrations of the indole analogues (Figure 6). All four compounds increased the survival of challenged brine shrimp larvae to over 80% at 100 μM (analogue 16) or 200 μM (analogues 19, 20 and 21). The survival of challenged brine shrimp larvae treated with indole did not reach this survival level at any of the concentrations tested, and complete mortality was observed at 200 μM of indole, confirming the toxicity of indole at this concentration.

**FIGURE 7:**
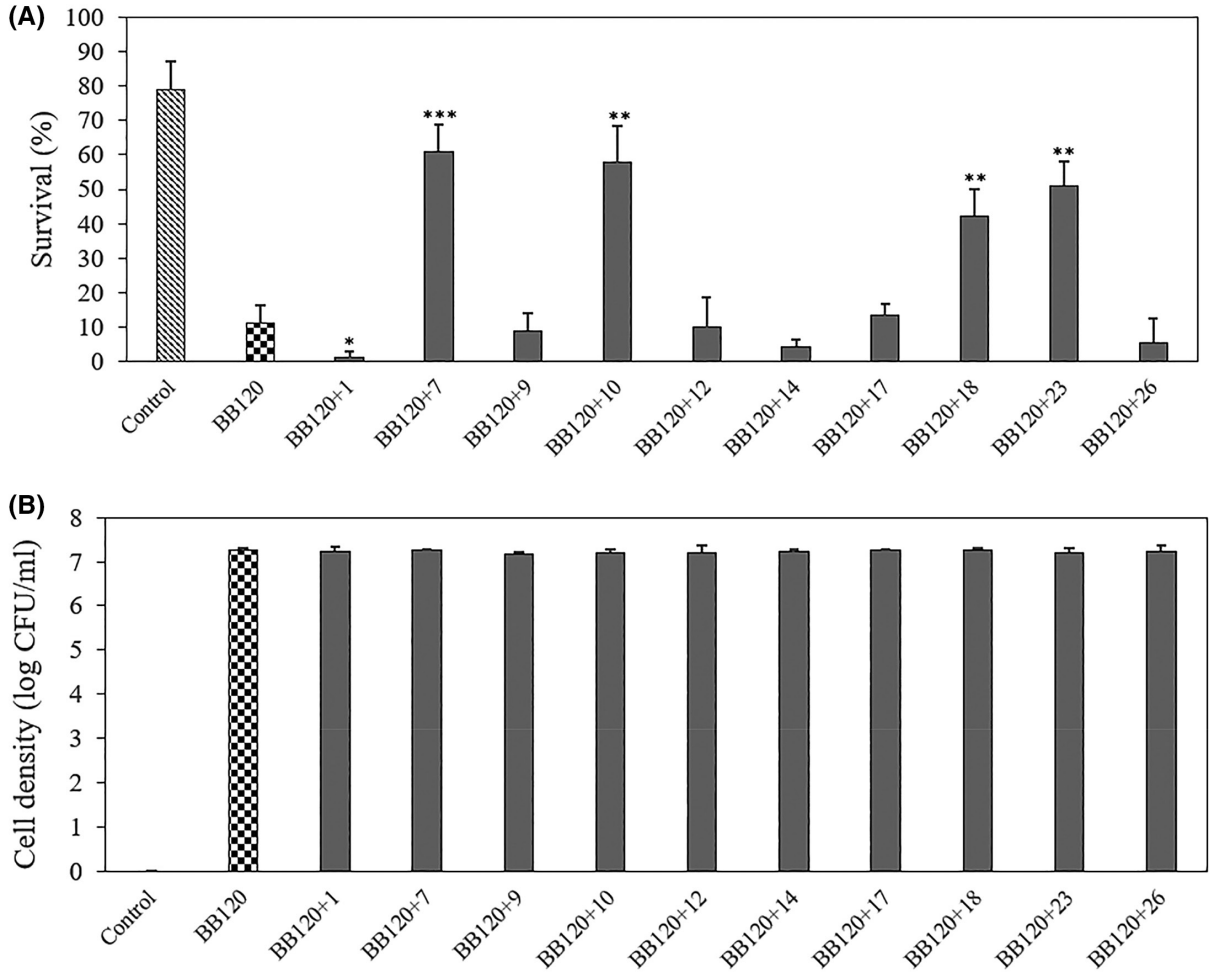
(A) Percent survival of brine shrimp (*Artemia franciscana*) larvae after 2 days of challenge with *V. campbellii* BB120 and 20 μM of selected indole analogues. *V. campbellii* BB120 and the indole analogues were added to the brine shrimp rearing water at the start of the experiment. (B) Density of *V. campbellii* BB120 with or without 20 μM of selected indole analogues in the brine shrimp larvae rearing water after 2 days of challenge. Error bars represent the standard deviation of three shrimp cultures. Control refers to brine shrimp cultures without addition of *V. campbellii BB120* that were treated in the same way as the other cultures. Asterisks indicate significant differences when compared to the treatment of BB120 without indole analogue (independent‐samples *T*‐test); *p < 0.05, **p < 0.01 and ***p < 0.001

**FIGURE 8:**
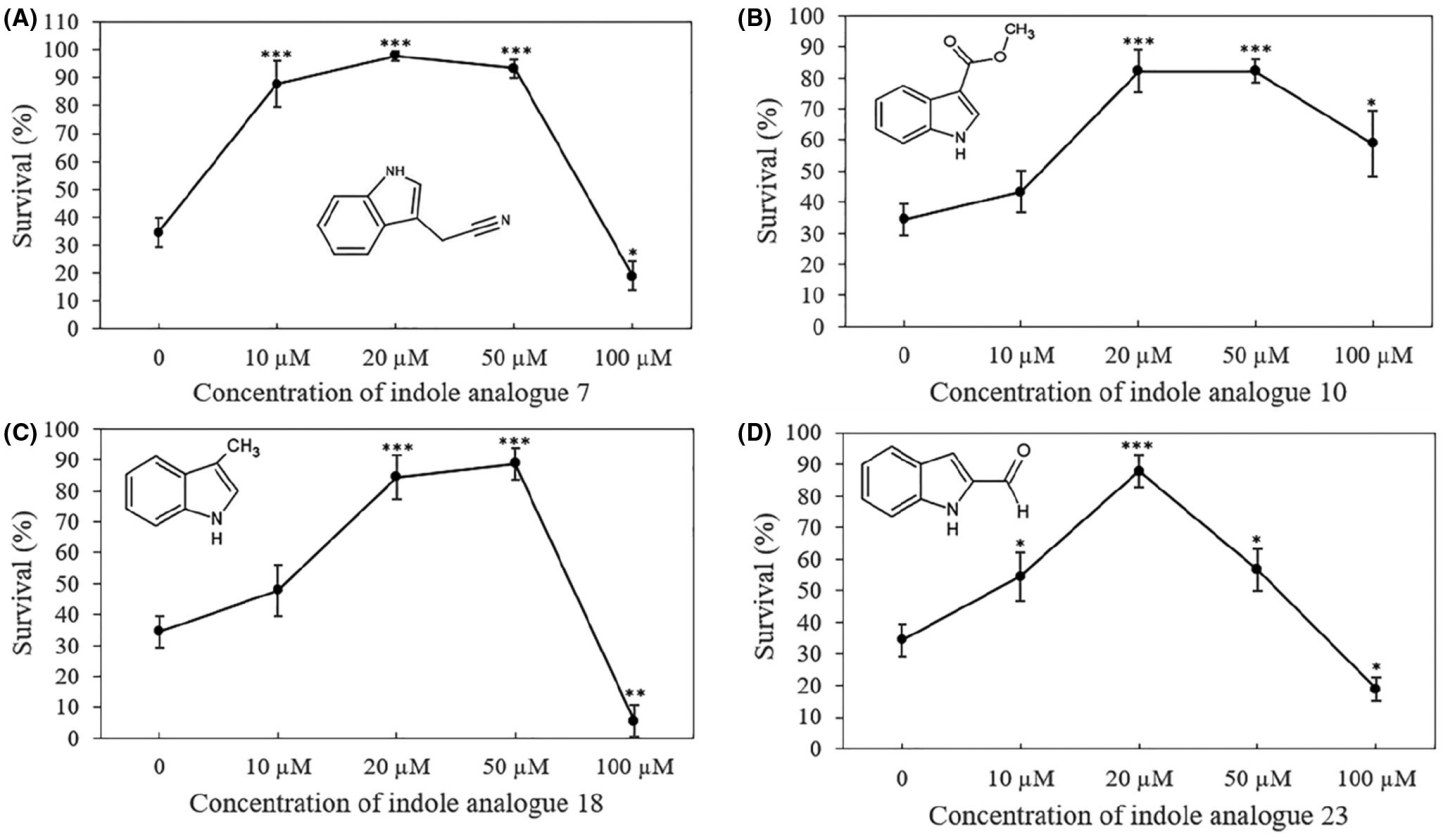
Percent survival of brine shrimp (*Artemia franciscana*) larvae after 2 days of challenge with *Vibrio campbellii* BB120 and different concentrations of indole analogues 7 (A), 10 (B), 18 (C), and 23 (D). Indole analogues were added to the brine shrimp rearing water at the start of the experiment. Error bars represent the standard deviation of three shrimp cultures. The survival of unchallenged larvae that were otherwise treated in the same way as challenged larvae was 94% ± 7%. Asterisks indicate significant differences when compared to the control treatment without indole analogue (independent‐samples *T*‐test); *p < 0.05, **p < 0.01 and ***p < 0.001

The results obtained with indole made us rethink that the low survival with the indole analogues added at 200 μM (i.e. for compounds 1, 7, 9, 10, 12, 14, 17, 18, 23 and 26) might be due to toxicity, and that we might have missed compounds with a high virulence inhibitory activity at lower concentrations. Therefore, we explored the impact of indole analogues on the virulence of *V. campbellii* towards brine shrimp larvae at a lower concentration of 20 μM. In this experiment, indole analogues 7 (indole‐3‐acetonitrile), 10 (methylindole‐3‐carboxylate), 18 (3‐methylindole) and 23 (indole‐2‐carboxaldehyde) significantly improved the survival of the brine shrimp larvae (Figure 7). Finally, we also explored the impact of different concentrations of these indole analogues on brine shrimp larvae challenged with *V. campbellii*. A high survival (>80%) was obtained at 10 μM or more of compound 7, and at 20 μM of the other three compounds (Figure 8). All four compounds showed toxicity at 100 μM (and also at 50 μM for analogue 23). On the basis of the results, IC50 values can be estimated to be between 50 and 100 μM for analogues 7, 18 and 23, and above 100 μM for analogue 10. Hence, for these most active analogues (excdpt for analogue 23), there is some safety margin between the active and toxic concentrations.

Indole analogues have been shown to interfere with virulence factor production of a wide range of bacterial pathogens, such as *E. coli* (Lee, Kim, Kim, et al., 2012), *S. marcescens* (Sethupathy et al., 2020), *P. aeruginosa* (Lee, Kim, Cho, et al., 2012), and *V. campbellii* (Yang et al., 2017). However, most of them have not been tested in vivo in animals challenged with the pathogen of interest. In the present study, 1‐methylindole (indole analogue 16), indene (indole analogue 19), 2,3‐benzofuran (indole analogue 20), thianaphthene (indole analogue 21), indole‐3‐acetonitrile (indole analogue 7), methyl indole‐3‐carboxylate (indole analogue 10), 3‐methylindole (indole analogue 18), and indole‐2‐carboxaldehyde (indole analogue 23) showed significant protective effect on brine shrimp larvae against *V. campbellii*. Indole‐3‐acetonitrile has been reported to decrease the biofilm formation of *E. coli* and *P. aeruginosa* at 100 mg/L (=640 μM) (Lee et al., 2011). In this study, indole‐3‐acetonitrile also inhibited the biofilm formation and motility of *V. campbellii*, and it improved the survival of brine shrimp larvae to 88% even at a much lower concentration of 10 μM. It has also been reported that 3‐methylindole (skatole) reduced biofilm formation of *E. coli* at 100 mg/L (=760 μM) (Choi et al., 2014). In this study, 3‐methylindole showed no effect on biofilm formation but decreased the motility of *V. campbellii*, and it improved the survival of brine shrimp larvae to 84% at a much lower concentration of 20 μM. The other six analogues that demonstrated protective effects for brine shrimp larvae against *V. campbellii* have not been previously reported to affect the virulence of any bacterial pathogen. The results obtained with analogues 19 (indene), 20 (2,3‐benzofuran) and 21 (thianapthene) are especially interesting as they suggest that these molecules, in which the nitrogen atom in the pyrrole moiety of indole is replaced by carbon, oxygen and sulphur, respectively, could also be used as a backbone for the design of indole analogues with antivirulence properties. Indeed, these compounds increased the survival of challenged brine shrimp larvae to over 80% and thus did not show toxicity at 200 μM (which is in contrast to what was observed for indole).

In conclusion, the present study demonstrates the abilities of indole analogues to protect brine shrimp larvae from infection caused by *Vibrio campbellii* without affecting bacterial growth. This is consistent with the concept of antivirulence therapy, which does not kill the pathogens but rather blocks virulence of the pathogens (Defoirdt, 2018). Eight indole analogues were found to increase the survival of brine shrimp larvae challenged with *V. campbellii* to high levels (>80%) when added in a preventive set‐up (i.e. together with the pathogen), while there was no protection offered in a curative set‐up (i.e. adding the analogues 1 day after addition of the pathogen). The most active analogue was indole‐3‐acetonitrile, which was active at 10 μM. Three other analogues – methylindole‐3‐carboxylate, 3‐methylindole and indole‐2‐carboxaldehyde – were active at 20 μM. Further, 1‐methylindole was active at 100 μM, and finally, the three indole analogues with a substitution in the pyrrole moiety – indene, 2,3‐benzofuran and thianaphthene – were active at 200 μM.

## AUTHOR CONTRIBUTIONS

**Shanshan Zhang:** Formal analysis (equal); funding acquisition (equal); investigation (equal); methodology (equal); validation (equal); visualization (equal); writing – original draft (equal). **Qian Yang:** Methodology (equal); supervision (equal); writing – review and editing (equal). **Tom Defoirdt:** Conceptualization (equal); funding acquisition (equal); methodology (equal); resources (equal); supervision (equal); writing – review and editing (equal).

## CONFLICT OF INTEREST

The authors declare that there are no conflicts of interest.

## Supporting information


Figure S1.
