# Supplementary material for: Indole analogues decreasing the virulence of Vibrio campbellii towards brine shrimp larvae
Source: Microb Biotechnol. 2022 Nov 5;15(12):2917–28. doi: 10.1111/1751-7915.14160 (PMC9733641; doi:10.1111/1751-7915.14160)

**Indole analogues decreasing the virulence of *Vibrio campbellii* towards brine shrimp larvae – Supplementary material**

Shanshan Zhang, Qian Yang, Tom Defoirdt

**Fig S1.** Growth of *V. campbellii* BB120 in LB_35_ medium supplemented with indole analogues at concentrations of 100 and 200 µM. The error bars represent the standard deviation of three independent experiments.


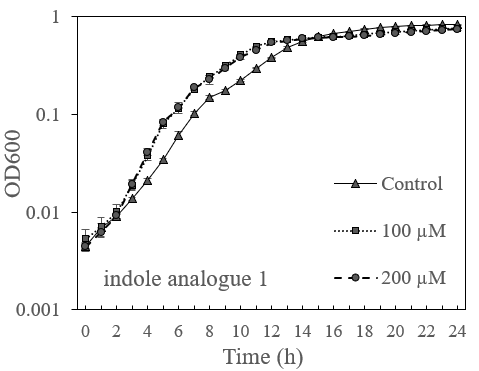


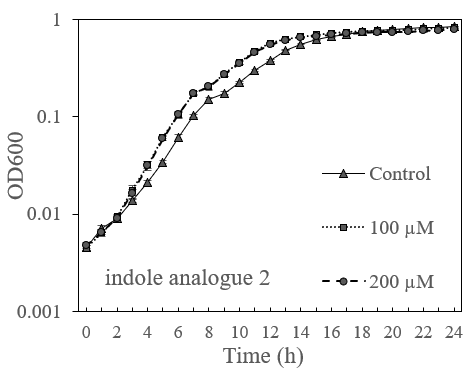


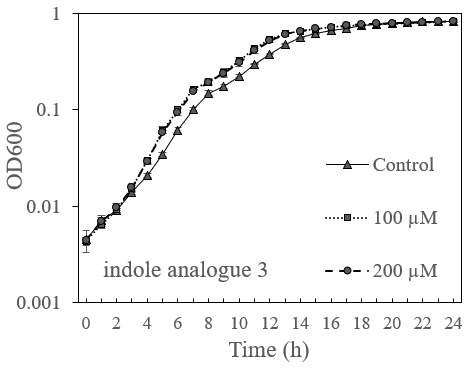


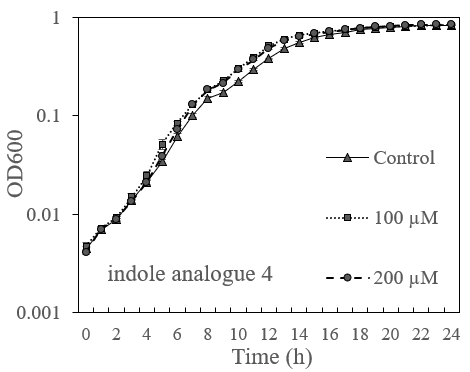


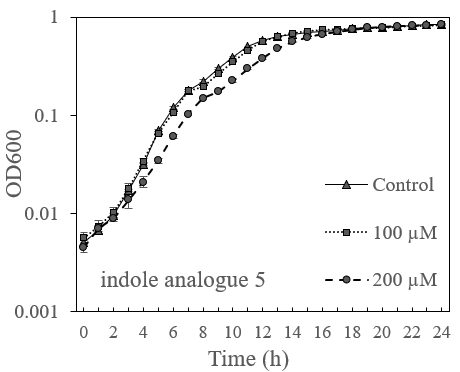


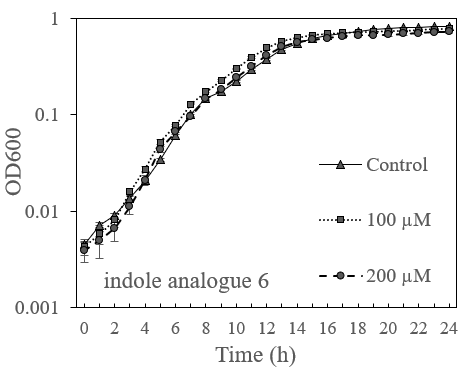


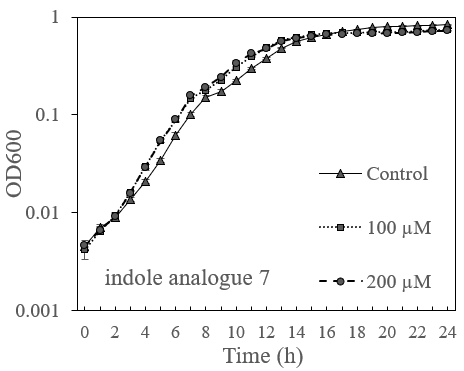


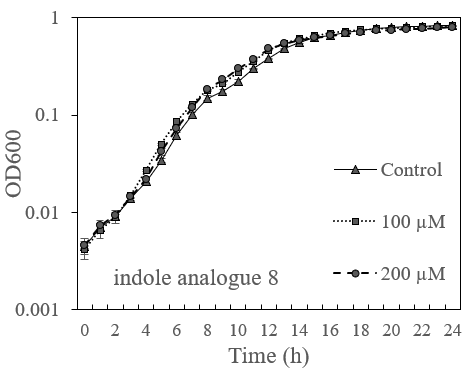


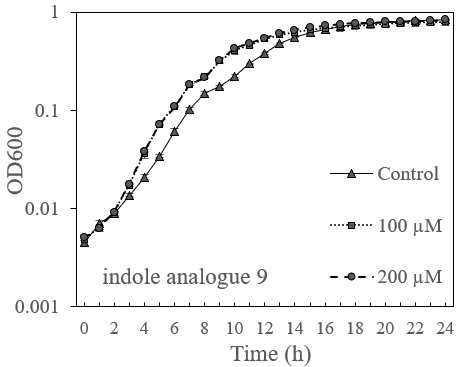


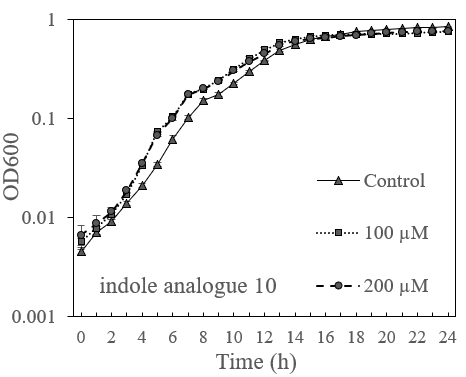


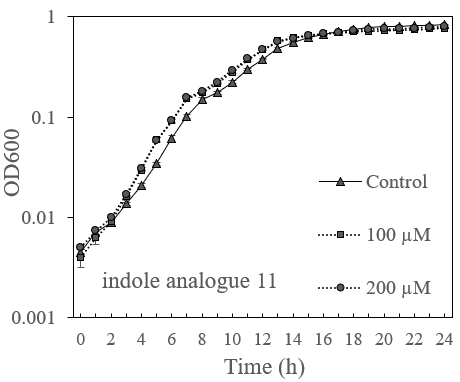


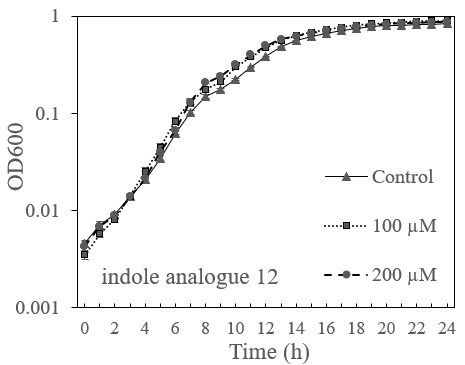


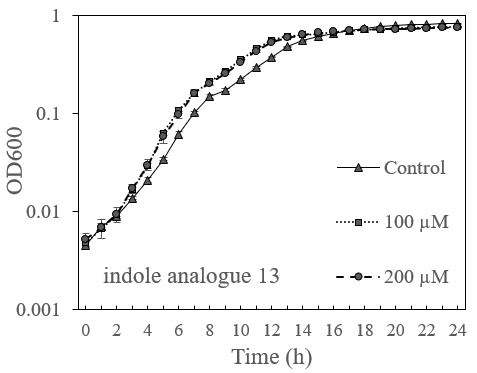


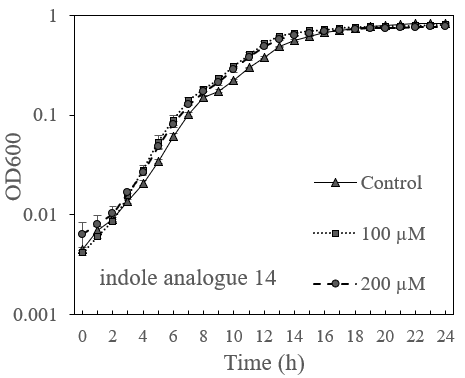


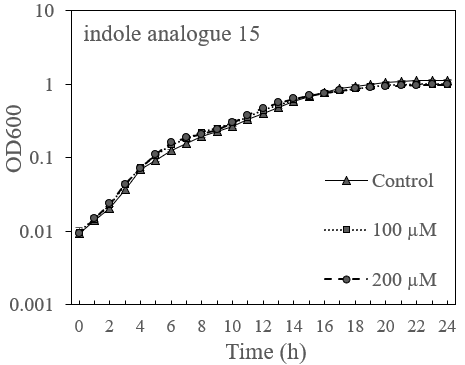


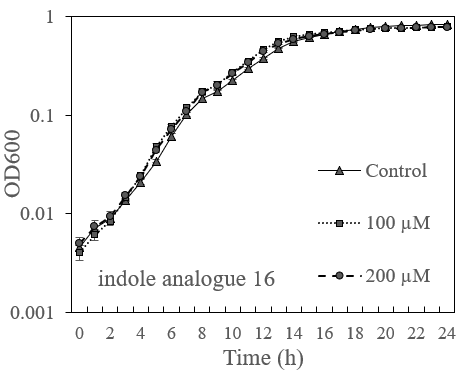


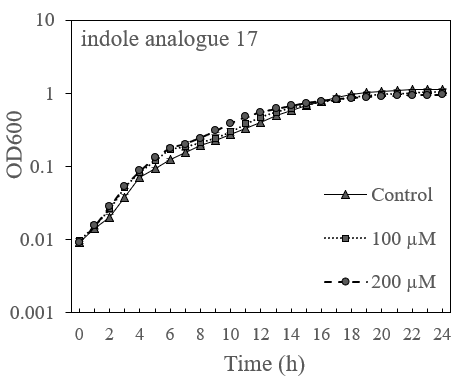


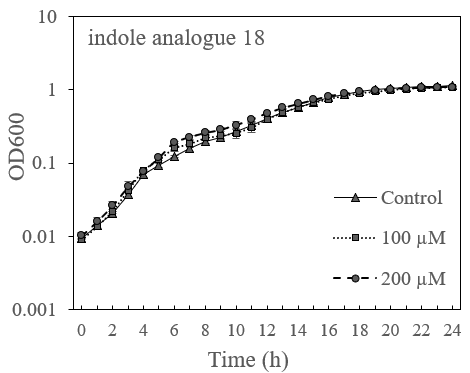


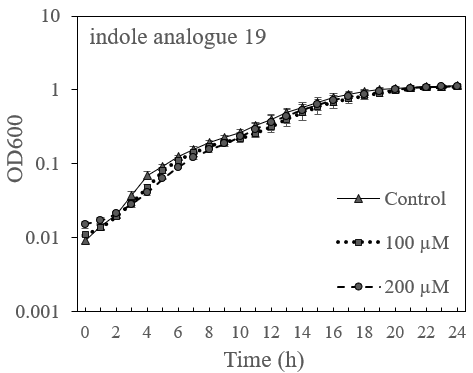


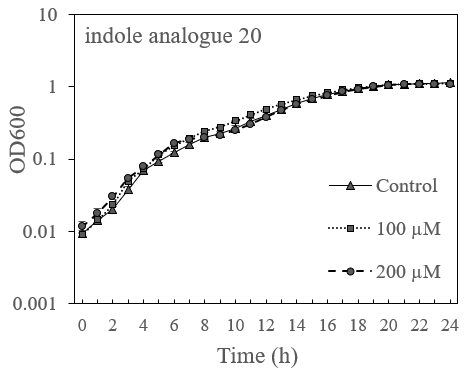


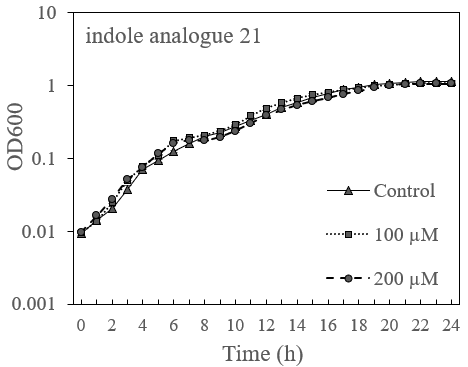


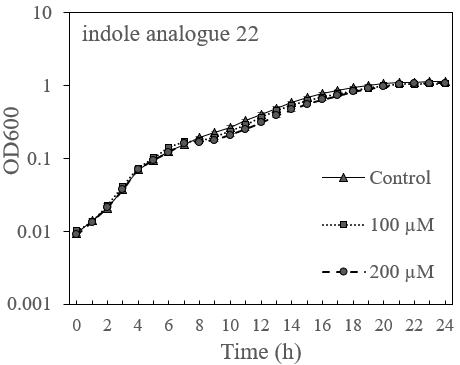


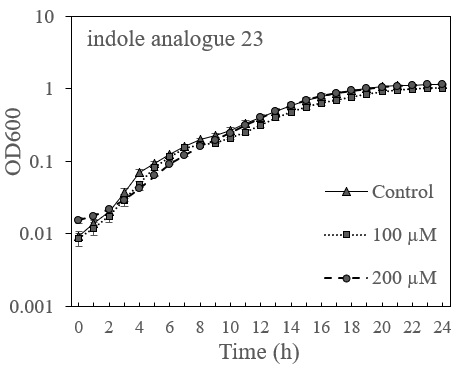


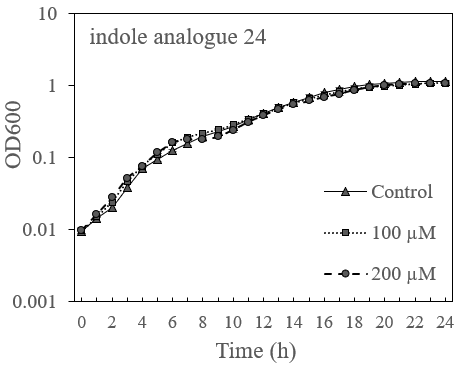


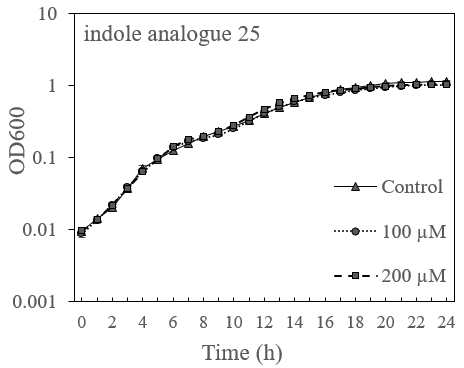


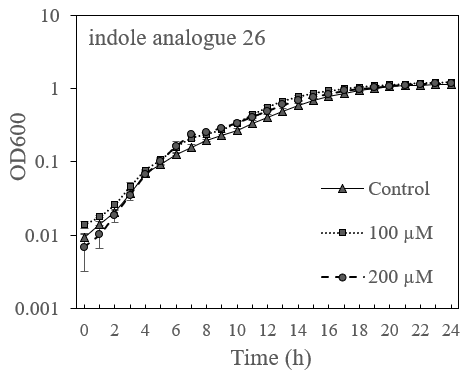

Supplement: Supplementary file 1 — Figure S1. [file MBT2-15-2917-s001.docx]
